# Association between Vitamin D Receptor Polymorphism and Susceptibility to Oral Lichen Planus and Oral Squamous Cell Carcinoma

**DOI:** 10.22038/IJORL.2024.73925.3489

**Published:** 2024-03

**Authors:** Nooshin Mohtasham, Farnaz Mohajertehran, Fahimeh Afzaljavan, Alieh Farshbaf, Kiumars Maraqehmoqadam, Maryam Tavakoliroodi, Majid Mirhashemi

**Affiliations:** 1 Oral and Maxillofacial Diseases Research Center, Mashhad University of Medical Sciences, Mashhad, Iran.; 2 Dental Research Center, Mashhad University of Medical Sciences, Mashhad, Iran.; 3 Clinical Research Development Unit, Imam Reza Hospital, Faculty of Medicine, Mashhad University of Medical Sciences, Mashhad, Iran.; 4 School of Dentistry, Mashhad University of Medical Sciences, Mashhad, Iran.; 5 Department of Oral and Maxillofacial Pathology, School of Dentistry, Mashhad University of Medical Sciences, Mashhad, Iran.

**Keywords:** ApaI, Squamous Cell Carcinoma of Head and Neck, Oral lichen planus, Mouth Neoplasms, Vitamin D receptor, rs7975232

## Abstract

**Introduction:**

Oral squamous cell carcinomas (OSCC) comprise 90-95% of oral cancers. Early diagnosis improved the survival rate of OSCC patients to 80–90%. Oral lichen planus (OLP) is a chorionic inflammatory disease with malignancy potential. The vitamin D receptor (VDR) plays a critical role in the pathogenesis of oral cancer. This study aimed to determine the association between VDR rs7975232 (Apa I) polymorphism and potential susceptibility to OLP and OSCC risks.

**Materials and Methods:**

In this prospective case-control study, a total of 120 blood samples were obtained from OSCC patients (n=29), OLP (n=50), and controls (n=40). VDR rs7975232 polymorphism was studied using the Polymerase Chain Reaction Restriction Fragment Length Polymorphism (PCR-RFLP) method. Statistical analysis was performed with SPSS Version 23 software. Data were expressed as means ± standard deviation (SD). Age, sex, allelic frequency, and genotyping were compared using the chi-square test. A p-value of less than 0.05 was regarded as statistically significant. The disease risk was estimated by Odds ratio (OR) with a 95% confidence interval.

**Results:**

A significant age difference was observed between the controls and the OSCC group (p=0.001). A significant difference was observed in Aa and aa genotypes compared with AA between OSCCs and controls. Moreover, dominant (p<0.001), additive (p<0.001), and allelic (p=0.001) models were different between groups.

**Conclusion:**

There was a positive association between rs7975232 VDR polymorphism and susceptibility to OSCC. More experimental evidence must reveal the possible association between rs7975232 and the risk of OLP in a larger cohort.

## Introduction

Oral cancer patients visit the clinic when they are in the late stage of the disease, and this causes increasing mortality. Oral cancer often manifests the signs of the clinical premalignant phase, there is sufficient time for early diagnosis to decrease morbidity and mortality (1). Oral squamous cell carcinomas (OSCC) are known as the most common malignant oral cancer that consists of 90-95% of oral neoplasms. Delayed diagnosis provides 5-year survival rates with poor prognosis and low survival rates. Early diagnosis improved the survival rate of OSCC patients to 80–90% (2). Although well-known causative factors such as smoking and alcohol drinking play pivotal roles in OSCC, the genetic background should not be overlooked. Tumor growth and development is a multi-step process and is affected by specific molecular pathways in different stages involving different genes. The changes in the sequence of these genes alter the function and impact the downstream targets of the cellular pathway.On the other hand, some of the changes mentioned do not influence gene roles but are associated with tumorigenesis, metabolic, and autoimmune diseases (3). Single nucleotide polymorphisms (SNPs) are genetic variants in every 100–300 human DNA sequence nucleotides that manifest in more than 1% of the population. Although SNPs do not disturb the function of the gene’s product, they can be applied as genetic markers in association studies (4).

The vitamin D receptor (VDR) gene is located on chromosome 12q13.11 and comprises two extensive promoter regions that can produce multiple tissue-specific transcripts. More than 200 single nucleotide polymorphisms (SNPs) are known in the VDR gene (5). 

Most studies evaluated the association between current VDR polymorphisms, including Apal (rs7975232), BsmI (rs1544410), Taql (rs731236), Fokl (rs10735810), and disease related to the VDR function (6). The Apal (rs7975232) polymorphism that is a candidate to apply in the current study is located on the intronic region of chromosome 12 (47871419 nt) VDR gene (c.1025-49 G > T) (1).Calcitriol or 1,25-dihydroxy vitamin D3 (1,25(OH)_2_D3) is an active metabolite vitamin D that plays an anti-tumor role by regulating cell proliferation and differentiation. 

**Fig 1:**
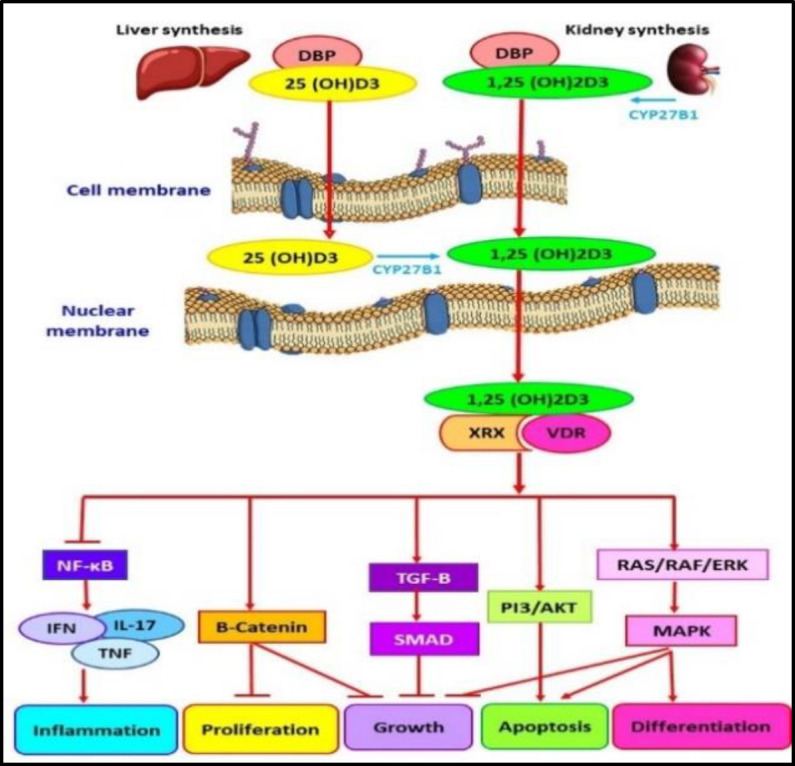
Calcitriol or 1,25-dihydroxy vitamin D3 (1,25(OH)2D3) affects cellular pathways involved in inflammation, proliferation, growth, apoptosis, and differentiation

When a cell begins malignant transformation, 1,25-dihydroxy vitamin D can induce apoptosis, inhibit angiogenesis, and help the cells survive. In this way, vitamin D regulates the function of genes related to the cell cycle, proliferation, inflammation, and apoptosis (Figure 1) (7). 

It was demonstrated that vitamin D receptor (VDR) polymorphisms can affect the interaction of 1,25-dihydroxyvitamin D and VDR and defect regulation of target downstream cellular pathways. These changes can be associated with bladder, colorectal, ovary, skin, renal cell carcinoma, prostate, and breast cancers (8). Oral lichen planus (OLP) is a chorionic inflammatory disease that appears following T-cell dysfunction. OLP is a benign disorder that affects oral mucosa, but 1.4% of lesions transformed into malignancies such as OSCC during seven years (9). It was estimated that 132 genes play a pivotal role in the etiopathogenesis of OLP that named "leader genes", VDR is introduced as one of the master genes that s2239185 and rs7975232 (Apa I) polymorphism were in correlated to OLP (10). It was demonstrated that genetic variants of VDR impact its interaction with vitamin D and change the downstream NF-κB pathway that is involved in the pathogenesis of OLP (11). It was reported that vitamin D decreased the expression of lncRNA LUCAT1, which regulated the growth and proliferation of OSCC by promoting of the mitogen protein kinase (MAPK) pathway. Also, LUCAT1 is more expressed in parallel to the increase of OSCC’s tumor size (12). It was reported that VDR plays a critical role in the pathogenesis of cutaneous melanoma. Thus, it can be applied as a prognostic marker and be considered for therapeutic approaches (13). Calcitriol up-regulates the cyclin-dependent kinase inhibitors (CDKI) and prevents cells from proliferation. Vitamin D levels and specific VDR polymorphisms were reported as essential factors in thyroid autoimmunity and cancer development (14). According to the crucial role of VDR in the pathogenesis of oral cancer, we decided to evaluate OLP patients -as a precancerous lesion- and OSCC -as malignant lesion- for the first time among Iranian population. Therefore, we aimed in this study to determine the association between VDR rs7975232 (Apa I) polymorphism and potential susceptibility to OLP and OSCC risks.

## Materials and Methods

*The study subjects*

In this prospective case-control study, a total of 120 blood samples were obtained from OSCC patients (n=29), OLP (n=50), and healthy control individuals (n=40). The age and sex matched in healthy controls. These samples were collected from the Department of Oral and Maxillofacial Pathology, School of Dentistry, Mashhad University of Medical Sciences, Omid and Quaem hospitals, Mashhad, Iran, from 2021 to 2022. Demographic, clinical, and medical information from all OLP and OSCC patients were registered. All subjects were collected, coded, and labeled as required.

In the present study, the inclusion criteria were a normal range of vitamin D serum levels (30-100 mg/dl) of all participants and histopathological confirmation of diagnosis in OLP and OSCC patients. All recruited subjects were >18 years old. All study participants were asked to fill out informed consent. The exclusion criteria were patients diagnosed with cancer or lichenoid reaction, autoimmune and metabolic diseases, and pregnancy. Individuals who used vitamin and mineral supplements or drugs affecting vitamin D levels were also eliminated. The samples with insufficient DNA quality and concentration were omitted from the study. The study was approved by the local Ethical Committee of the Mashhad University of Medical Sciences (IR. MUMS. DENTSIRY. REC.1400.037). The genetic material remaining after analysis has been stored in the DNA bank of the Department of Oral and Maxillofacial Pathology, School of Dentistry, Mashhad University of Medical Sciences, Mashhad, Iran.

*DNA extraction*

DNA was isolated using FavorPrep^TM^ Blood Genomic DNA Extraction Mini Kit (Cat.No.: FABGK001) following the manufacturer’s recommendations and standardized procedures. Extracted DNA qualified by 1.5% agarose gel. The DNA quantification and purity were evaluated by the absorption of 260/280 and 260/230 nm ratio wavelength using a NanoDrop® 2000 spectrophotometer. DNA samples with an average of 50-100 ng/µl concentration and 1.5-2 optimal density (OD) showed a single sharp band (722 bp) in agarose gel selected for the following molecular steps and stored at -20 °C.

*Polymerase Chain Reaction Restriction Fragment Length Polymorphism (PCR-RFLP) and VDR Genotyping*

**Fig 2:**
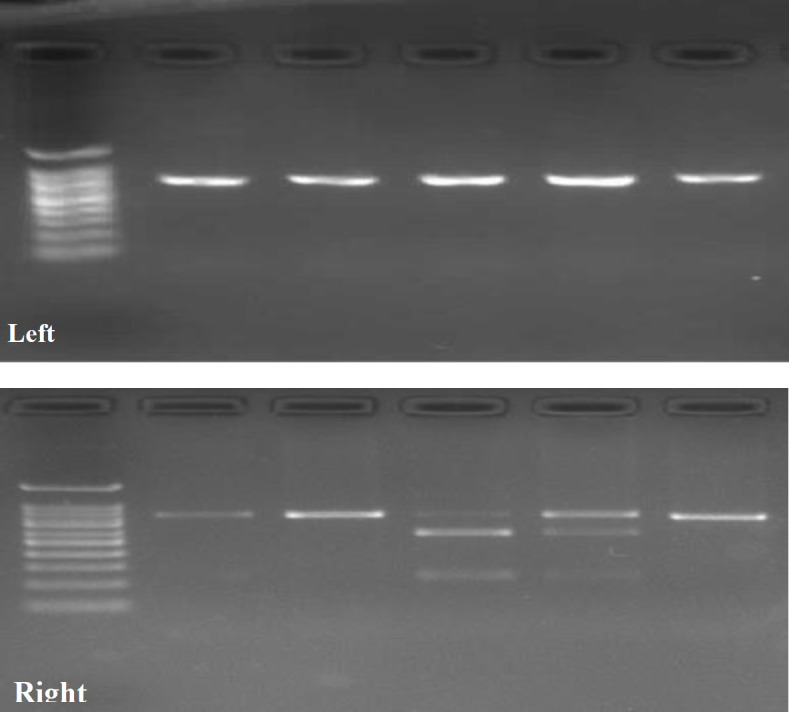
Left) Gel electrophoresis image of PCR products of VDR gene with 722 bp length; Right) Gel electrophoresis image of the PCR products after digestion with ApaI restriction enzymes. Genotypes were determined as AA (722 bp), Aa (722, 509, 213 bp), or aa (213 bp) for rs7975232 polymorphism as homozygous major, heterozygous, and homozygous minor, respectively

Detection of the VDR rs7975232 (Apa I) polymorphism was carried out using the conventional Polymerase Chain Reaction Restriction Fragment Length Polymorphism (PCR-RFLP) method with forward 5' CAGAGCATGGACAGGGAGCAA 3' and reverse 5' GCAACTCCTCATGGCTGAGGT CTC 3' primers to amplify desired DNA region of VDR gene. The reaction was performed in 20 µl total volume, including 10 µl of 2X Taq PCR Master Mix (Pars Tous, Iran), 1 µl forward and 1 µl reverse primers, 4 µl DNA template, and 4 µl nuclease-free water. The thermal conditions were pre-incubation at 95 °C for 5 min, and 40 cycles at 95 °C for 45 sec (denaturation), 62 °C for 45 sec (annealing), 72 °C for 45 sec (extension), and finalize extension at 72 °C for 5 min. The recommended protocol for digestion of PCR products directly after amplification was 10 µl of PCR product, 18 µl of nuclease-free water, 2 µl of 10X Buffer B, and 2 µl of restriction enzyme ApaI (Thermo Scientific, Ref.: ER1411). The mixture was incubated at 37 °C for 16 h. Then, the samples were incubated at 65°C for 20 min for enzyme inactivation. Next, they run on agarose gel 1.5% for evaluation of genotype. To validate the PCR process, we applied positive control per reaction. Genotypes were determined as AA (722 bp), Aa (722, 509, 213 bp), or aa (213 bp) for ApaI polymorphism considered as three genotype classes including homozygous major, heterozygous, homozygous minor, respectively (Figure 2).

*Data Analysis*

Statistical analysis was performed with the SPSS Version 23 statistic software package. Data were expressed as means ± standard deviation (SD). 

Age, sex, allelic frequency, and genotyping were compared using the chi-square test. A p-value of less than 0.05 was regarded as statistically significant. The disease risk was estimated by Odds ratio (OR) with a 95% confidence interval.

## Results

 The characteristics of the study population are summarized in Table 1. The study population included 40 healthy controls, 50 OLP cases, and 29 OSCC patients. 

 The mean age was 43.20±14.67, 47.04±15.42, and 54.21±12.00 in the control, OLP, and OSCC groups. A significant age difference was observed between the controls and the OSCC group (p=0.001). The distribution of sex was similar between groups.

**Table 1:** The study population characteristics

**Characteristics**		**Healthy**	**OLP**	**P-value**	**OSCC**	**P-value**
Age		43.20±14.67	47.04±15.42	0.234	54.21±12.00	0.001
Sex	Male	19 (47.5%)	20 (40%)	0.525	16 (55.2%)	0.628
	Female	21 (52.5%)	30 (60%)		13 (44.8%)	

Most OSCC patients belonged to grade II and stage I of the disease. In addition to 15 (51.7%) lymph node-positive cases, metastasis was observed in 13 OSCC subjects (44.8%). Considering position, 16 patients (55.2%) developed the disease in the tongue. In the OLP group, 28 patients (56%) indicated the disease in the buccal mucosa. The detailed clinicopathological features are reported in Table 2.

**Table 2:** Clinicopathological characteristics of the study population

**Group**	**Characteristics **		**Number **	**Percent **
OSCC	Grade	1	9	31.0
		2	16	55.2
		3	4	13.8
	Stage	1	15	51.7
		2	11	38.0
		3	3	10.3
	Metastasis	No	16	55.2
		Yes	13	44.8
	Lymph node	No	14	48.3
		Yes	15	51.7
	Position	Tongue	16	55.2
		Mouth	5	17.2
		Cheek	7	24.1
		Supraglottic	1	3.4
OLP	Site	Tongue	7	14.0
		Palate	2	4.0
		Mouth	8	16.0
		Gum	5	10.0
		Buccal mucosa	28	56.0

The distribution of rs7975232 genotypes was similar between the controls and the OLP group. Further analysis revealed a lack of association between rs7975232 and OLP in dominant (p=0.217), recessive (p=0.247), additive (p=0.701), and allelic (p=0.103) models. Considering OSCC subjects, a significant difference was observed in Aa and aa genotypes compared with AA between OSCCs and controls. Moreover, dominant (p<0.001), additive (p<0.001), and allelic (p=0.001) models were different between groups. 

However, the distribution of genotypes was similar between OSCC and control groups in the recessive model. Similar results were observed in adjusted analyses for age. Results are shown in Table 3.

**Table 3:** Genetic models of rs7975232 in association with OLP and OSCC

**Genetic model**		**Healthy**	**OLP**	**p**	**OR (95% CI)**	**OSCC**	**P**	**OR (95% CI)**	**p**_Adj_	**OR**_Adj_ ** (95% CI)**
Genotypes	AA	26 (65.0%)	26 (52%)		Ref.	3 (11.5%)		Ref.		Ref.
	Aa	9 (22.5%)	13 (26%)	0.475	1.44 (0.53-3.96)	18 (69.2%)	<0.001	17.33 (4.11-73.03)	<0.001	22.45 (4.40-114.59)
	aa	5 (12.5%)	11 (22%)	0.194	2.20 (0.67-7.22)	5 (19.2%)	0.014	8.67 (1.55-48.49)	0.012	12.48 (1.74-89.70)
Dominant	AA	26 (65%)	26 (52%)		Ref.	3 (11.5%)		Ref.		Ref.
	Aa+aa	14 (35%)	24 (48%)	0.217	1.71 (0.73-4.03)	23 (88.5%)	<0.001	14.24 (3.63-55.88)	<0.001	19.25 (4.04-91.79)
Recessive	AA+Aa	35 (87.5%)	39 (78%)		Ref.	21 (80.8%)		Ref.		Ref.
	aa	5 (12.5%)	11 (22%)	0.247	1.97 (0.62-6.24)	5 (19.2%)	0.459	1.67 (0.43-6.44)	0.371	2.00 (0.44-9.08)
Additive	AA+aa	31 (77.5%)	37 (78%)		Ref.	8 (30.8%)		Ref.		Ref.
	Aa	9 (22.5%)	13 (26%)	0.701	1.21 (0.46-3.21)	18 (69.2%)	<0.001	7.75 (2.54-23.65)	0.001	8.75 (2.49-30.73)
Allelic	A	61 (76.3%)	65 (65%)		Ref.	24 (46.2%)		Ref.		Ref.
	a	19 (23.8%)	35 (35%)	0.103	1.73 (0.89-3.34)	28 (53.8%)	0.001	3.75 (1.77-7.93)	<0.001	1.07 (1.04-1.10)

Further analyses indicated no association between rs7975232 genotypes and clinicopathologic features of OSCC, including stage, grade, lymph node status, metastasis, and position. These findings were repeated for all genetic models.

## Discussion

In the current study, 40 healthy controls, 50 OLP, and 29 OSCC patients participated. A significant age difference was observed between the controls and the OSCC group. The distribution of VDR rs7975232 (ApaI) genotypes was statistically different between OSCC and control groups in the dominant, additive, and allelic models.

The involvement of VDR polymorphisms in oral cancer development has been shown in several studies. Shen et al. evaluated 177 OLP patients and 207 healthy controls for eight VDR polymorphisms in the Chinese Han population. They reported that VDR Fok I and ApaI polymorphisms can impact OLP susceptibility (15). Compared to our study, they assessed more VDR SNPs in a larger population with different target ethical people. Especially since we did not find a statistically significant association between VDR ApaI polymorphism and OLP. However, we studied OSCC as a common malignancy type of oral cancer in addition to OLP with one polymorphism (ApaI).

The other VDR polymorphisms related to oral cancer were studied. Nigam et al. compared 300 controls to 230 precancerous oral patients, including 70 leukoplakia, 90 oral submucous fibrosis, 70 OLP, and 72 oral cancer patients (16). Compared to our study, they studied a larger population with the other types of precancerous oral lesions. Furthermore, they evaluated the Indian population and applied Taq 1 to assess polymorphism. They reported an association between VDR (Taq1) polymorphism, oral precancerous lesions, and oral cancer (16).

It was reported that there was a positive correlation between ApaI VDR polymorphism and other cancers. In a meta-analysis study, Laczmanski et al. evaluated 26 studies, including 5657 controls and 5113 patients with lung, neck, head, esophageal, and oral cancers (17). They evaluated FokI, ApaI, TaqI, and BsmI VDR polymorphisms to find an association between these candidates' VDR polymorphisms and mentioned tobacco-related cancers. They reported that the occurrence of the "t" allele in the TaqI VDR polymorphism decreased tobacco-related cancer risk by 17% (17). Gnagnarella et al. revealed the outcome of their systematic review study which was strong associations between ApaI VDR polymorphism and prostate, breast, and colorectal cancers. They considered different ethical heterogeneity in 176 independent studies. They highlighted the regulatory role of some genetic variations in the VDR gene (18). In a meta-analysis, Rao et al. evaluated three case-control articles with 728 HBV-related hepatocellular carcinomas (HCC) participants and 920 HBV-healthy control individuals. They aimed to study VDR polymorphisms and the risk of HBV-related HCC. They proposed that the “f” allele of Fok I polymorphism can be a risk factor for HCC in HBV-infected patients and can apply as a predictive factor for monitoring HCC in HBV-infected patients (19). Serrano et al. studied 73 independent articles during a meta-analysis that included 35525 cancer patients and 38675 healthy control individuals. They studied TaqI, ApaI, and Cdx2 VDR polymorphisms to assess the association between the risk of cancers and mentioned SNPs. They reported that Cdx2 (gg) polymorphism strongly correlated with a 12% increased risk for all cancers (20).

By the VDR's pivotal role in a wide variety of biological processes, it was revealed that ApaI VDR polymorphism was associated with clinical manifestation and liver disease progression in Vietnamese HBV-infected patients (21). It was reported a significant correlation between VDR polymorphism (Fok1, Bsm1) and the risk of type 2 diabetes mellitus (T2DM). This study evaluated 156 T2DM patients and 145 healthy individuals in the Egyptian population (22).

Among the Iranian population, there was no report like the current study to compare VDR polymorphisms between premalignant (OLP) and malignant (OSCC) lesions. Some of the research studies investigated the association between VDR polymorphisms and cancer in the Iranian population; however, there are some controversial reports: In 2022, Kazemi et al. compared 161 breast cancer cases with 150 healthy controls among Iranian women. They demonstrated that FokI (rs2228570), BsmI (rs1544410), and ApaI (rs7975232) polymorphisms had more frequency in patients than in the healthy control. In addition, they reported FokI, BsmI, and ApaI of VDR polymorphisms associated with the risk of breast cancer in Iranian women (23). However, Matini et al. in 2020 reported only VDR ApaI and TaqI polymorphisms associated with the risk of breast cancer. Their sample size and applied technique were similar to the Kazemi et al. study (24). For the first time, Hoseinkhani et al. in 2021 evaluated different VDR polymorphisms, including Fok1, ApaI, BsmI, and TaqI among 99 gastric cancers in comparison to 100 controls in the Iranian Kurdish population of Kermanshah. They reported a positive association between the distribution of FokI genotypes and gastric cancer risk (25). In 2020, Ramezi et al. investigated VDR FokI, BsmI, and Tru9I polymorphisms in 40 medullary thyroid cancer (MTC) patients and 40 controls by PCR sequencing. They revealed a strong correlation between Tru9I polymorphism and prevalency of MTC (26). In 2018, Moossavi et al. compared VDR FokI and TaqI polymorphisms among 100 colorectal cancer (CRC) patients and 100 controls in South Khorasan of Iran. They reported a positive correlation between ff genotype and f allele of FokI SNP with CRC which can increase the sucepibility and risk of South Khorasan population to CRC (27). 

In addition to cancer studies, there were reports about the association between VDR polymorphism and different diseases in the Iranian population: in 2023, Mahmoudi et al. manifested homozygous genotypes of VDR ApaI (aa) and FokI (ff) polymorphisms increased risk of brucellosis disease 53 folds more than wildtypes (AA and FF) (28). Other Iranian study populations showed VDR Fok-I and Taq-I polymorphisms had a significant an association with the risk of multiple sclerosis (MS) in the Kurdish population (29), BsmI had association with incidence of coronary artery disease (CAD) in the southern Iranian population (30), TaqI had an association with type 1 diabetes mellitus (TIDM) (31), and Fok I associated with Crohn disease's susceptibility (32). The level of vitamin D and VDR expression can affect the tumorigenesis process. For example, Grimm et al.’s study results in 191 OSCC patients showed decreased-VDR expression and improved tumor relapse which can be considered an independent prognostic factor. They disclosed that co-expression of CD44^+^ cancer stem cells and VDR may affect therapeutic approaches following adjuvant chemoprevention using 1,25-(OH)2D3 or its analogs (33). Polymorphism of the other genes manifested in relation to oral cancer. Nigam et al. showed a protective effect of DNA repair gene polymorphism, including XRCC3 and NBS1, on oral submucous fibrosis (OSMF), OLP, as well as oral cancer (34). In another study, they demonstrated that the polymorphism of N-acetyl transferases 1 (NAT1) decreases the risk of oral lesions, while the NAT2 polymorphism potential of oral cancer (35). They showed polymorphisms of Xerodermapigmentosum group D (XPD) (A/C) and group G (XPG) (G/C) genes influenced preoral cancer as well as oral cancer progress and clinical manifestation (36). It was reported that gene expression changes during the tumorigenesis process by different regulatory molecules such as microRNAs (37). 

It was demonstrated that miR-122 reduced VDR expression to increase the excessive apoptosis by targeting NF-κB to promote the destruction of the epithelial barrier and cause the pathogenesis of OLP (38). miR-26a/b expression plays a vital role in the pathogenesis of OLP by inhibiting apoptosis by targeting Protein Kinase C because it is down-regulated in tissue biopsies, serum, and saliva in patients. Thus, unchanged VDR binding sites, besides the high expression of VDR, can reduce the inflammatory response in oral keratinocytes (39). In accordance with the previous studies, the distribution of VDR polymorphisms such as rs7975232 (Apa I) are different among various ethnical populations, and the result of the association study between rs7975232 and OLP and OSCC can be affected by sample sizes. Since the VDR altered expression and its accurate interaction with vitamin D active downstream cellular pathways that target genes are involved in proliferation, differentiation, inflammation, apoptosis, and angiogenesis, VDR can be considered a pivotal factor in the biopathogenesis of many diseases and tumors.We proposed designing more comprehensive studies (like cohorts) in larger populations that comprise different ethnical populations and evaluating more SNPs and haplotypes as markers for the association between them and potentially oral premalignant lesions (such as OLP) and oral malignant lesions.

## Conclusion

There was a positive association between rs7975232 VDR polymorphism and susceptibility to OSCC. More experimental evidence needs to reveal the possible association between VDR gene polymorphisms and the risk of OLP in a larger cohort. Early diagnosis helps OSCC patients reduce mortality, and the clinical follow-up of OLP can prevent malignancy transformation.
